# Relationship between digital health literacy and self-assessment of health among older adults in Xuzhou City: based on the mediating effect of health self-management

**DOI:** 10.3389/fpubh.2026.1744492

**Published:** 2026-03-18

**Authors:** Zhaohui Qin, Huangying Shen, Yan Xu, Hongan Zhang, Xiliang Li, Zhiwen Zhao, Yichen Li, Wenhao Huang

**Affiliations:** 1 The Second Clinical Medical School, Xuzhou Medical University, Xuzhou, Jiangsu, China; 2 School of Management, Xuzhou Medical University, Xuzhou, Jiangsu, China; 3 School of Public Health, Xuzhou Medical University, Xuzhou, Jiangsu, China

**Keywords:** digital health literacy, health self-management, mediation analysis, older adults, self-assessed health

## Abstract

**Background:**

With the global rise in aging populations and the proliferation of digital technologies, digital health literacy has emerged as a critical determinant of older adults’ ability to access health information and enhance their health outcomes. This study investigates the complex interplay and mechanisms linking digital health literacy, health self-management, and self-assessed physical health among older adults in Xuzhou, China.

**Methods:**

In November 2024, a questionnaire-based survey was conducted among adults aged 60 and older in urban communities of Xuzhou City, Jiangsu Province, China. The questionnaire assessed general demographic characteristics, self-assessed physical health, the Chinese version of the Digital Health Literacy Scale, and the Health Self-Management Scale. A total of 1,005 participants were included, comprising 47% males and 53% females, with a mean age of 67.6 years. Data were analyzed using SPSS 23 software.

**Results:**

The mean scores for self-assessed physical health, digital health literacy, and health self-management were 3.41 ± 0.83, 24.75 ± 11.74, and 159.72 ± 21.24, respectively. Statistically significant differences were observed in these scores across age groups, marital status, pre-retirement occupation, number of medications, living arrangement, monthly disposable income, and daily internet use (all *p* < 0.05). Digital health literacy, health self-management, and self-assessed physical health were significantly positively correlated (all *p* < 0.01). Digital health literacy positively predicted self-assessed physical health (*β* = 0.007, *p* = 0.041). Health self-management fully mediated the relationship between digital health literacy and self-assessed physical health, with an indirect effect of 0.005 (95% Bootstrap *confidence interval* 0.003–0.007), accounting for 71.4% of the total effect.

**Conclusion:**

The findings indicate that digital health literacy positively influences health self-management among older adults. Moreover, through the mediating role of health self-management, digital health literacy contributes to improvements in health behaviors and overall health status among older adults.

## 1 Introduction

China is experiencing one of the fastest aging population processes in the world. The defining demographic trend of the 21st century is the global aging population. According to the World Health Organization (WHO), the global population aged 60 and older is projected to reach 1.4 billion by 2030 and 2.1 billion by 2050 (1). In 2024, China’s population aged 60 and older reached 310.31 million, comprising 22% of the total population, of which those aged 65 and older accounted for 15.6% (2). By United Nations standards, China has entered a moderately aging society. The chronic health problems of older adults that come with the extension of life expectancy have become prominent. How to maintain good health, address the issue of population aging, and achieve healthy aging has become an important public health issue faced by China and even the world.

With advancements in Internet technology and the rise of the digital era, health information and management have become increasingly digitized. Digital technologies are being integrated into medical and healthcare systems, driving innovation in the industry. The WHO’s 2020–2025 Global Digital Health Strategy emphasizes the importance of leveraging digital technologies to improve human health and achieve the vision of health for all (3). The advancement of digital technology, particularly in promoting healthy aging among older adults, holds significant potential (4). The WHO’s 2023–2030 Regional Digital Health Action Plan further highlights digital health literacy as a key factor in achieving universal health coverage (5), underscoring the need to enhance individuals’ digital literacy skills to improve health and wellbeing (6). Digital health literacy is defined as an individual’s ability to seek, access, understand, and evaluate health information from electronic sources and apply this knowledge to address or resolve health-related issues (7). It significantly influences health outcomes by shaping how individuals search for and utilize online health information (8). Individuals with higher digital health literacy are more likely to actively seek and acquire digital health resources, improve their health based on this information (9), adopt regular physical exercise habits (10), maintain balanced dietary practices (11), and report higher self-perceived health, mental wellbeing, and self-efficacy (12). Moreover, they exhibit a lower risk of developing chronic diseases (13). Consequently, digital health literacy is a critical determinant of health outcomes (14). These positive effects provide a foundation for empowering older adults to effectively manage their health.

The high prevalence of chronic non-communicable diseases underscores the critical role of health self-management in maintaining the health of older adults. Enhancing self-management capabilities in this population is a viable strategy to address the challenges of population aging and the rising incidence of chronic diseases (15). Health self-management involves older adults actively monitoring and managing their physical functions through biological, psychological, and sociological interventions (16). Research indicates that self-management capabilities significantly contribute to maintaining overall health in older adults (17) and are strongly correlated with subjective wellbeing and reduced depression (18). A key determinant of effective self-management is digital health literacy. With the increasing adoption of digital health management tools, digital health literacy is essential for disseminating information that enhances self-management and improves health outcomes (19). Studies demonstrate that older adults with higher digital health literacy exhibit more positive health behaviors and stronger self-management capabilities (20, 21). Concerning chronic disease management, leveraging digital technologies to enhance self-management can improve quality of life and slow disease progression (22). Higher digital health literacy enables patients to better understand their diagnoses, leading to improved self-management behaviors (23). For example, cancer patients may use digital health technologies to support self-management, addressing their ongoing and complex healthcare needs (24). Similarly, individuals with type 2 diabetes utilize digital tools to enhance self-management, optimize metabolic control, and prevent acute and chronic complications (25). Conversely, inadequate digital health literacy is associated with reduced health behaviors, deteriorated health outcomes, and diminished self-management capabilities (26).

**Figure 1:**
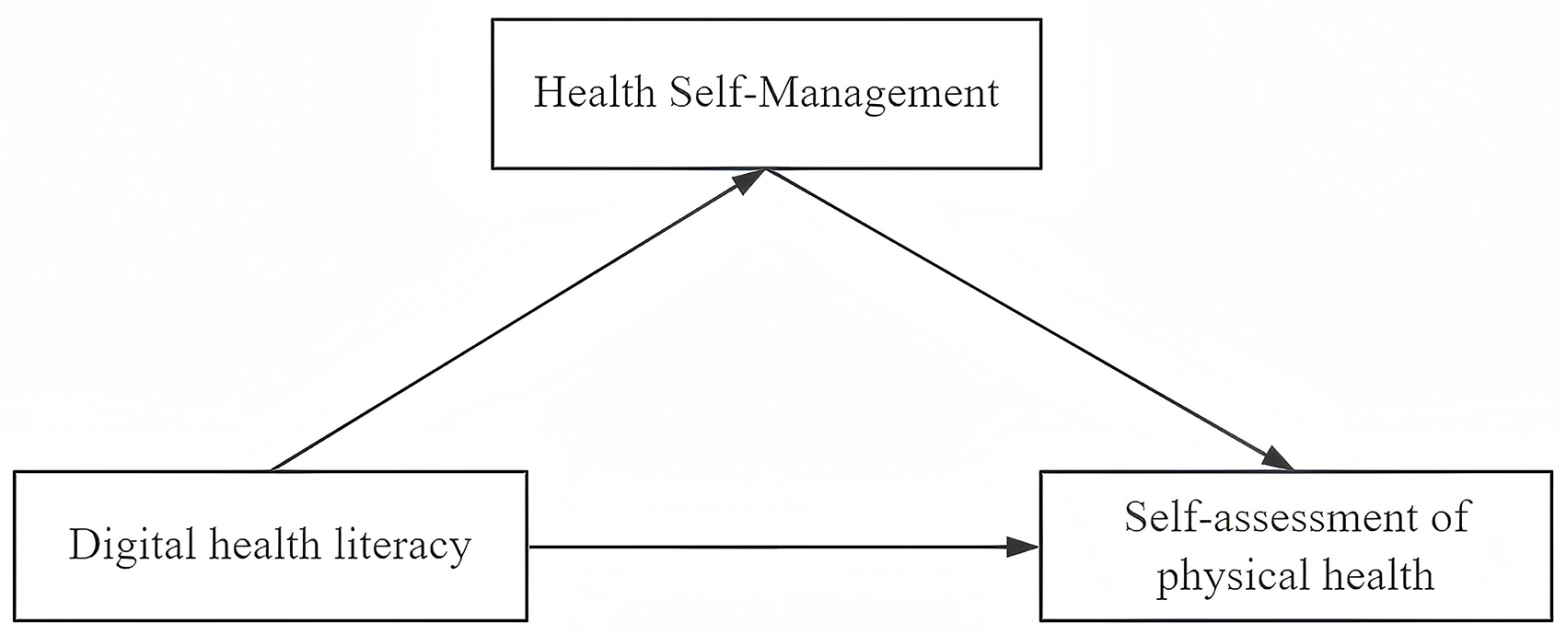
Hypothetical model.

Although existing studies have, respectively, confirmed the importance of digital health literacy and health self-management, However, the precise pathways through which digital health literacy influences physical health outcomes among older adults, particularly the mediating role of health self-management, remain underexplored. Accordingly, this study aims to examine the relationship between digital health literacy and self-assessed physical health among older adults in China, along with the mediating mechanism of health self-management. To address these objectives, the study proposes two hypotheses to frame its conceptual model. First, digital health literacy, health self-management, and self-assessed physical health among older adults are positively correlated. Second, health self-management mediates the association between digital health literacy and self-assessed physical health. Figure 1 illustrates the study’s theoretical framework.

## 2 Materials and methods

### 2.1 Research participants

This cross-sectional study was conducted in urban communities of Xuzhou City, China, in November 2024. A multi-stage stratified random cluster sampling method was employed to select participants. First, based on the 2023 statistical yearbook from the Xuzhou Municipal Bureau of Statistics, the seven urban administrative regions of Xuzhou City were classified into three economic levels, including high, medium, and low, according to their gross domestic product. One administrative region was randomly selected from each level. Next, two to four communities were randomly selected from each chosen administrative region, resulting in a total of 10 communities as sampling points. All adults aged 60 and older residing in these communities were invited to participate in a questionnaire survey. Inclusion criteria were age ≥60 years, residence in urban communities of Xuzhou City for at least one year, provision of informed consent for voluntary participation and possessing basic communication skills. Exclusion criteria included those assessed by investigators as having severe audio-visual impairments, obvious cognitive dysfunction that affects communication, or mental abnormalities that prevent normal communication. Unable to operate basic digital devices independently or with assistance.

The study was conducted by trained investigators from the research team. Before the investigation began, the investigator explained the purpose of this study to the participants. After obtaining their informed consent, a face-to-face questionnaire survey was conducted among them in various communities of Xuzhou City. The questionnaire is asked item by item by the investigator. Participants answer orally, and the investigator records the answers on the spot. The completion time for each questionnaire is approximately 15 to 20 min. A total of 1,050 questionnaires were distributed, with 1,005 valid questionnaires returned, yielding an effective response rate of 95.71%. The study received ethical approval from the Ethics Committee of Xuzhou Medical University (Approval Number: XZHMU-2021037).

### 2.2 Measure

The questionnaire collected data on gender, age, marital status, prevalence of chronic diseases, educational attainment, sociodemographic characteristics, and monthly disposable income. Additionally, it evaluated self-assessed physical health, digital health literacy, and health self-management.

#### 2.2.1 Self-assessment of physical health

Self-assessed physical health serves as an indicator of the physical health status of older adults. Research indicates a strong correlation between older adults’ physical health and their self-assessed health, making self-assessment a reliable measure for evaluating functional health in daily life (27). Physical health was assessed using the question, “How would you rate your health condition?” Responses were recorded on a 5-point Likert scale, ranging from 1 (“very poor”) to 5 (“very good”). Higher scores indicate more favorable self-assessed health.

#### 2.2.2 Digital health literacy

Digital health literacy was measured using the Chinese version of the eHealth Literacy Scale (eHEALS), developed by Guo et al. (28). This scale has been validated and is widely used among older adults in China (29). It consists of eight items, each rated on a 5-point Likert scale from 1 (“strongly disagree”) to 5 (“strongly agree”), with total scores ranging from 8 to 40. Higher scores reflect greater digital health literacy. In this study, the Cronbach’s alpha for the scale was 0.98, indicating excellent internal consistency.

#### 2.2.3 Health self-management

Health self-management of older adults was assessed using the Adult Health Self-Management Ability Scale (AHSMSRS), developed by Huang et al. (30). This scale consists of 38 items across three dimensions: self-management behavior (14 items), self-management environment (14 items), and self-management cognition (10 items). Each item is rated on a 5-point Likert scale, with total scores ranging from 38 to 190. Higher scores indicate greater health self-management capabilities among older adults. In this study, the Cronbach’s alpha for the scale was 0.96, demonstrating excellent internal consistency.

### 2.3 Covariates

Based on the research design, this study included factors that might affect the self-assessment of physical health, digital health literacy, and health self-management of older adults as covariates, including sociodemographic characteristic variables, health-related variables, and digital behavior variables. Among them, sociodemographic characteristics include gender, age, marital status, living arrangement, educational level, pre-retirement occupation, and monthly disposable income. Variables related to health status include the prevalence of chronic diseases and number of medications. Digital behavior variables include daily internet use.

### 2.4 Data analysis

Statistical analyses were conducted using SPSS version 23. Continuous variables, including self-assessed physical health, digital health literacy, and health self-management, were reported as mean ± standard deviation (SD). Categorical variables were presented as frequencies and percentages (*n*, %). Independent *t*-tests and analysis of variance (ANOVA) were used to compare differences in scores for self-assessed physical health, digital health literacy, and health self-management across sociodemographic characteristics among older adults. Pearson correlation analysis was employed to examine the relationships among self-assessed physical health, digital health literacy, and health self-management. A mediation model was constructed using the PROCESS Macro (31), with digital health literacy as the predictor variable, health self-management as the mediator, and self-assessed physical health as the outcome variable. The significance of the indirect effect was evaluated using 5,000 bootstrap samples, with significance indicated if the 95% confidence interval excluded zero. Two-sided *p*-values were used for all statistical analyses.

## 3 Results

### 3.1 Participants’ sociodemographic characteristics

A total of 1,005 older adults from urban communities in Xuzhou City participated in this study, comprising 472 males (47.0%) and 533 females (53.0%). Participants’ ages ranged from 60 to 90 years, with a mean age of 67.60 years (SD = 6.73). Of the participants, 881 (87.7%) were married, while 124 (12.3%) were unmarried, widowed, or divorced. Regarding educational attainment, 256 (25.5%) had primary school education or below, 384 (38.2%) had completed junior high school, and 365 (36.3%) had attained senior high school or higher. In terms of pre-retirement occupation, 284 (28.3%) were farmers, 373 (37.1%) were enterprise employees, 196 (19.5%) were government or public institution employees, 63 (6.3%) were self-employed, and 75 (7.4%) were freelancers. For monthly disposable income, 408 (40.6%) reported ≤2,000 RMB, 311 (31.0%) reported 2,001–4,000 RMB, 178 (17.7%) reported 4,001–6,000 RMB, and 108 (10.7%) reported ≥6,000 RMB. Regarding living arrangements, 49 (4.8%) lived alone, 459 (45.7%) lived with their spouse, and 486 (48.4%) lived with their children. Additionally, 702 participants (69.9%) reported having chronic diseases. In terms of medication use, 265 participants (26.4%) reported not taking any medication, 285 (28.4%) reported taking one medication, and 455 (45.2%) reported taking two or more medications. Furthermore, 776 participants (77.2%) reported using the Internet daily.

### 3.2 Differences in self-assessment of physical health, digital health literacy and health self-management scores among older adults

The mean scores for self-assessed physical health, digital health literacy, and health self-management were 3.41 ± 0.83, 24.75 ± 11.74, and 159.72 ± 21.24, respectively. Significant differences in these scores were observed across age groups, marital status, pre-retirement occupation, number of medications, living arrangement, monthly disposable income, and daily internet use (all *p* < 0.05). Educational attainment did not significantly affect self-assessed physical health scores, nor did gender or chronic disease status significantly influence digital health literacy scores (see Table 1 for detailed comparisons).

**Table 1:** Comparison of self-assessed physical health, digital health literacy, and health self-management scores across sociodemographic characteristics.

Variable	*N* (%)	Self-assessment score for physical health			Digital health literacy score			Health self-management score		
		M ± SD	*t/F*	*p*	M ± SD	*t/F*	*p*	M ± SD	*t/F*	*p*
Gender			−2.795	0.005		1.062	0.288		−3.275	0.001
Man	472 (47)	3.33 ± 0.85			25.17 ± 11.65			157.40 ± 22.22		
Woman	533 (53)	3.48 ± 0.81			24.38 ± 11.82			161.78 ± 20.13		
Age (years)			15.208	0.000		61.409	0.000		43.983	0.000
60–69	665 (66.2)	3.51 ± 0.82			27.26 ± 10.48			163.40 ± 18.64		
70–79	276 (27.5)	3.19 ± 0.82			21.21 ± 12.51			155.17 ± 22.96		
≥80	64 (6.4)	3.30 ± 0.75			13.94 ± 10.73			141.23 ± 25.39		
Marital status			3.793	0.000		7.527	0.000		8.119	0.000
Marriage	881 (87.7)	3.34 ± 0.84			25.77 ± 11.43			161.70 ± 20.29		
Non-marriage	124 (12.3)	3.15 ± 0.72			17.52 ± 11.40			145.67 ± 22.60		
Education level			2.440	0.088		71.405	0.000		12.425	0.000
Primary and lower	256 (25.5)	3.39 ± 0.86			19.02 ± 11.20			155.07 ± 21.63		
Junior high school	384 (38.2)	3.35 ± 0.80			23.95 ± 11.99			159.20 ± 21.22		
High school level or above	365 (36.3)	3.48 ± 0.83			29.61 ± 9.67			163.53 ± 20.31		
Pre-retirement occupation			2.397	0.036		25.399	0.000		10.506	0.000
Farmer	284 (28.3)	3.38 ± 0.93			18.86 ± 11.44			154.93 ± 22.12		
Enterprise employee	373 (37.1)	3.38 ± 0.81			27.03 ± 11.36			161.44 ± 20.26		
Government or public institution employee	196 (19.5)	3.57 ± 0.76			29.05 ± 10.13			166.94 ± 18.61		
Self-employed	63 (6.3)	3.22 ± 0.68			23.41 ± 11.32			152.25 ± 21.21		
Freelancer	75 (7.4)	3.34 ± 0.77			25.13 ± 10.34			155.94 ± 23.38		
Other	14 (1.4)	3.50 ± 0.82			27.07 ± 11.78			163.57 ± 16.06		
Monthly disposable income (RMB)			3.988	0.008		39.420	0.000		12.135	0.000
≤2,000	408 (40.6)	3.37 ± 0.91			20.99 ± 11.96			156.35 ± 22.15		
2,001–4,000	311 (31.0)	3.33 ± 0.80			24.89 ± 11.50			159.35 ± 21.51		
4,001–6,000	178 (17.7)	3.54 ± 0.73			28.48 ± 9.87			162.21 ± 18.40		
≥6,001	108 (10.7)	3.57 ± 0.74			32.42 ± 8.30			169.40 ± 17.80		
Living arrangement			4.423	0.004		4.267	0.005		11.307	0.000
Living alone	49 (4.8)	3.22 ± 0.91			22.00 ± 12.73			155.9 ± 22.31		
Living with spouse	459 (45.7)	3.47 ± 0.82			26.12 ± 11.41			163.5 ± 20.21		
Living with children	486 (48.4)	3.38 ± 0.81			23.77 ± 11.81			156.9 ± 21.39		
Other	11 (1.1)	2.72 ± 0.46			22.63 ± 12.45			141.2 ± 22.15		
Chronic diseases			8.068	0.000		1.846	0.065		5.027	0.000
Yes	702 (69.9)	3.27 ± 0.79			24.30 ± 11.97			164.79 ± 17.29		
No	303 (30.1)	3.72 ± 0.84			25.79 ± 11.15			157.53 ± 22.39		
Number of medications			84.284	0.000		22.710	0.000		35.459	0.000
0	265 (26.4)	3.90 ± 0.75			26.06 ± 11.58			166.5 ± 18.08		
1	285 (28.4)	3.37 ± 0.75			27.68 ± 11.20			162.5 ± 22.04		
≥2	455 (45.2)	3.13 ± 0.78			22.14 ± 11.62			153.9 ± 20.91		
Daily internet use			5.459	0.000		71.590	0.000		12.988	0.000
Yes	776 (77.2)	3.48 ± 0.82			29.69 ± 8.44			164.5 ± 18.36		
No	229 (22.8)	3.15 ± 0.79			8.00 ± 0.00			143.5 ± 22.32		

### 3.3 Correlation between self-assessment of physical health, digital health literacy, and health self-management among older adults

Pearson correlation analysis was used to examine the relationships among self-assessed physical health, digital health literacy, and health self-management in older adults. As shown in Table 2, digital health literacy was positively correlated with self-assessed physical health (*r* = 0.185, *p* < 0.01) and health self-management (*r* = 0.496, *p* < 0.01). Self-assessed physical health was also positively correlated with health self-management (*r* = 0.300, *p* < 0.01).

**Table 2:** Correlations among self-assessed physical health, digital health literacy, and health self-management in older adults.

Variable	Self-assessment of physical health	Digital health literacy	Health self-management
Self-assessment of physical health	1		
Digital health literacy	0.185**	1	
Health self-management	0.300**	0.496**	1

** indicates *p* < 0.01.

### 3.4 Mediating effect of health self-management on digital health literacy and self-assessment of physical health among older adults

#### 3.4.1 Common method deviation test

Harman’s single-factor test was conducted to evaluate common method bias. Eight factors with eigenvalues greater than 1 were extracted without rotation, and the first factor explained 39% of the variance, below the 40% threshold (32). Thus, no significant common method bias was present.

#### 3.4.2 Mediating effects of health self-management on digital health literacy and self-assessment of physical health among older adults

In this study, self-assessed physical health was used as the dependent variable, while digital health literacy was treated as the independent variable and health self-management was examined as a potential mediator. Prior to constructing the mediation model, multicollinearity diagnostics were performed for all independent variables and covariates. The results indicated that the variance inflation factor (VIF) values for all variables were below 5 and tolerance values exceeded 0.1, suggesting no significant multicollinearity in the model (see Table 3). Subsequently, PROCESS Macro was used for mediating effect analysis, controlling for gender, age, marital status, chronic disease status, educational attainment, pre-retirement occupation, medication usage, living arrangement, monthly disposable income, and daily internet use. In Table 4, Model 1 designated self-assessed physical health as the dependent variable and digital health literacy as the independent variable. Model 2 assigned health self-management as the dependent variable, with digital health literacy as the independent variable. Model 3 designated self-assessed physical health as the dependent variable, including both digital health literacy and health self-management as independent variables. In Models 1 and 2, digital health literacy had a significant positive effect on self-assessed physical health (*b* = 0.007, *p* = 0.042) and health self-management (*b* = 0.740, *p* < 0.001). In Model 3, health self-management significantly predicted self-assessed physical health (*b* = 0.007, *p* < 0.001), while digital health literacy showed no significant effect, indicating full mediation by health self-management.

**Table 3:** Multicollinearity test.

Variable	Tolerance	VIF
Gender	0.866	1.155
Age	0.732	1.367
Marital status	0.851	1.175
Chronic diseases	0.763	1.311
Number of medications	0.739	1.352
Education level	0.667	1.499
Pre-retirement occupation	0.925	1.081
Monthly disposable income	0.658	1.519
Living arrangement	0.946	1.057
Daily internet use	0.370	2.702
Digital health literacy	0.340	2.944
Health self-management	0.673	1.487

**Table 4:** Mediating effects of health self-management on the relationship between digital health literacy and self-assessed physical health among older adults.

Variable	Model 1: self-assessment of physical health			Model 2: health self-management			Model3: self-assessment of physical health		
	*b*	*SE*	*p*	*b*	*SE*	*p*	*b*	*SE*	*p*
Gender	0.114	0.051	0.025	4.626	1.181	0.001	0.083	0.051	0.103
Age	−0.057	0.046	0.212	−2.249	1.062	0.019	−0.040	0.045	0.375
Marital status	−0.165	0.078	0.034	−7.707	1.804	0.000	−0.113	0.078	0.146
Education level	−0.029	0.038	0.433	−1.097	0.868	0.206	−0.022	0.037	0.554
Pre-retirement occupation	−0.010	0.020	0.614	−0.521	0.465	0.263	−0.007	0.020	0.740
Monthly disposable income	0.074	0.029	0.011	1.506	0.674	0.025	0.064	0.029	0.027
Living arrangement	−0.030	0.038	0.430	−3.314	0.878	0.002	−0.007	0.038	0.844
Chronic diseases	−0.149	0.059	0.012	−1.385	1.377	0.315	−0.140	0.059	0.018
Number of medications	−0.311	0.033	0.000	−3.875	0.769	0.000	−0.285	0.033	0.000
Daily internet use	0.025	0.093	0.792	−0.335	2.165	0.877	0.027	0.092	0.771
Digital health literacy	0.007	0.003	0.042	0.740	0.077	0.000	0.002	0.003	0.607
Health self-management							0.007	0.001	0.000
*R^2^*	0.179			0.327			0.199		
*F*	19.622 (*p* < 0.001)			43.928 (*p* < 0.001)			20.565 (*p* < 0.001)		

#### 3.4.3 Test on the mediating effect of older adults’ health self-management on digital health literacy and self-assessment of physical health

The bootstrap method was used to evaluate the significance of the mediation effect of health self-management. Table 5 presents the results of this mediation analysis. Digital health literacy significantly predicted self-assessed physical health among older adults (*β* = 0.007, *p* = 0.041). Additionally, health self-management fully mediated the relationship between digital health literacy and self-assessed physical health (*β* = 0.005, *p* = 0.000), with the indirect effect accounting for 71.4% of the total effect (effect size = 0.005).

**Table 5:** Bootstrap test for mediating effect of health self-management.

Effect	Path	*β*	SE	*p*	LLCI	ULCI
Direct effect c’	Digital health literacy→Self-assessment of physical health	0.002	0.003	0.607	−0.005	0.008
Indirect effect	Digital health literacy→Health self-management→Self-assessment of physical health	0.005	0.001	0.000	0.003	0.007
Total effect c	Digital health literacy→Self-assessment of physical health	0.007	0.003	0.041	0.0003	0.013

*β* = coefficient of effect, SE = standard error, LLCI = lower limit of the confidence interval, ULCI = upper limit of the confidence interval.

## 4 Discussion

This study aimed to examine the relationships among digital health literacy, health self-management, and self-assessed physical health among older adults in China, as well as the mechanisms influencing these relationships. The findings confirmed positive correlations between self-assessed physical health, digital health literacy, and health self-management among older adults. Significant differences were observed in self-assessed physical health, digital health literacy, and health self-management scores across age groups, marital status, and pre-retirement occupation, number of medications, living arrangement, monthly disposable income, and daily internet use (*p* < 0.05). Additionally, health self-management was found to fully mediate the relationship between digital health literacy and self-assessed physical health among older adults. The hypotheses proposed in the study, specifically Hypothesis 1 and Hypothesis 2, have been successfully validated.

### 4.1 The digital health literacy level of older adults

The digital health literacy level of older adults in our study sample (mean: 24.75) was intermediate, falling below Park’s research on community-dwelling adults aged 65 and older in the United States (mean: 27.63) (33), yet surpassing Li et al.’s investigation on urban and rural older adults in Jinan City, China (mean: 17.56) (9). These discrepancies may stem from our participants being older adults in urban communities. Several studies confirm that digital health literacy of older adults in rural areas of China is significantly lower than that of their urban counterparts (34), primarily attributed to uneven development of Internet access between urban and rural regions (35). The educational attainment of older adults in rural areas is often low, coupled with limited awareness regarding active learning and economic resources, which contribute to a diminished focus on personal health. Access to electronic devices and the Internet is also often inadequate in rural settings. The COVID-19 pandemic may have influenced the enhancement of digital health literacy among older adults, evident in the accelerated integration of digital health technologies into medical services and increased reliance on these technologies (36). The Internet played a vital role in delivering health-related information and services to older adults, helping address physical and mental health challenges (37). Although digital health literacy levels in our study increased compared to previous studies, they did not reach the passing level of ≥32 points (38), indicating a substantial gap in the effective use of digital tools to access and apply health information. This finding also suggests that basic digital literacy alone may be insufficient to directly translate into measurable health benefits.

### 4.2 The direct impact of digital health literacy on the self-assessment of physical health among older adults

The results highlight a significant association between digital health literacy and self-assessed physical health among older adults, consistent with previous research (39, 40), which shows that higher levels of digital health literacy are associated with improved health outcomes (41). Theoretically, older adults with greater digital health literacy can access a wider range of health-related information and better understand their personal health through electronic resources, which supports improved management of chronic diseases and greater participation in preventive health practices (42). However, this study identified a relatively weak correlation between digital health literacy and self-assessed physical health, consistent with findings by Brørs et al. (43). This weak correlation may be due to older adults’ trust in the health information they access. Trust plays a critical role in the acceptance and use of health information (44). Older adults often rely more on traditional media and healthcare providers than on online sources for health information. Although the Internet is a valuable tool for providing health-related knowledge and encouraging healthy behaviors (45), its open and less-regulated nature results in varying information quality. Current regulatory frameworks are insufficient, allowing the dissemination of unverified health information, which leads to skepticism among older adults regarding the accuracy and reliability of digital health resources. This skepticism may reduce their effective use of digital health information, thereby limiting the direct impact of digital health literacy on their health outcomes. This phenomenon suggests that digital health literacy may mainly influence specific health behaviors rather than directly affect health outcomes.

### 4.3 The mediating role of health self-management

This study further confirms that health self-management plays a significant mediating role in the association between digital health literacy and self-rated physical health among older adults. The mediating effect accounted for 71.4% of the total effect, which is higher than that reported in previous studies (67.4%) (46). These findings indicate that the influence of digital health literacy on overall health among older adults is primarily achieved through the behavioral pathway of health self-management. Previous studies have shown that digital technology empowerment provides both technical support and social resources for active health management in older populations, thereby enhancing self-management capacity and promoting health equity (47, 48). Strong health self-management skills are associated with improved quality of life and functional status. Through behaviors such as enhanced self-monitoring, appropriate medication use, and healthy dietary practices, older adults can shift from a “passive response” to an “active health” approach, ultimately contributing to better overall health outcomes. However, in the context of the deep integration of Internet and healthcare services, merely enhancing digital skills of older adults is insufficient. Effective empowerment of their health self-management behaviors through systematic interventions is critical to fostering sustainable health practices and advancing overall health levels.

### 4.4 Limitations

This study explores the relationships among digital health literacy, health self-management, and self-assessed physical health among older adults, aligning with the global rise in digital technology and the challenges of population aging. The findings provide novel insights into improving the physical wellbeing of older adults and promoting healthy aging. However, several limitations must be acknowledged. The cross-sectional design prevents establishing temporal relationships among digital health literacy, health self-management, and self-assessed physical health, limiting causal inferences. Furthermore, participants in this study were recruited from urban communities in Xuzhou City. Given the substantial urban–rural disparities in Internet infrastructure, digital health literacy, and access to healthcare services in China, the findings primarily reflect the characteristics of older adults living in urban settings and may have limited generalizability to non-urban populations. Future research should consider incorporating mixed urban–rural samples or conducting multi-regional comparative studies to more comprehensively examine the mechanisms through which digital health literacy influences health outcomes among older adults across diverse social environments. Third, although potential confounding factors were controlled for, the influences on self-assessed physical health and digital health literacy among older adults remain complex and multifaceted. In particular, self-rated health is a subjective measure that may be influenced by psychological factors, personal expectations, and cultural norms, potentially introducing reporting bias. Additionally, unmeasured variables, such as older adults’ utilization of health services, may affect outcomes. While these limitations may impact the study’s representativeness and applicability, the findings offer valuable theoretical and practical insights for enhancing the physical health of older adults.

## 5 Conclusion

This study established a relationship among digital health literacy, health self-management, and self-assessed physical health among older adults. The results confirm positive correlations among digital health literacy, health self-management, and self-assessed physical health. Health self-management mediates the relationship between digital health literacy and self-assessed physical health. Improving digital health literacy among older adults may encourage more positive health behaviors and thereby contribute to better health outcomes. Therefore, governmental bodies should recognize the importance of digital health literacy and health self-management, advocate for integrating digital technology into health promotion efforts for older adults, enhance their digital health literacy and self-management skills, and ultimately improve their overall health status.

## Data Availability

The original contributions presented in the study are included in the article and/or supplementary material. If you have any questions, please contact the corresponding author.
